# The role of protease inhibitors on the remineralization of demineralized dentin using the PILP method

**DOI:** 10.1371/journal.pone.0188277

**Published:** 2017-11-28

**Authors:** Hamid Nurrohman, Karina M. M. Carneiro, John Hellgeth, Kuniko Saeki, Sally J. Marshall, Grayson W. Marshall, Stefan Habelitz

**Affiliations:** 1 Department of Preventive and Restorative Dental Sciences, University of California, San Francisco, San Francisco, CA, United States of America; 2 Missouri School of Dentistry and Oral Health, A.T. Still University, Kirksville, Missouri, United States of America; 3 Faculty of Dentistry, University of Toronto, Toronto, Ontario, Canada; 4 Thermo Fisher Scientific, San Jose, CA, United States of America; Institute of Materials Science, GERMANY

## Abstract

Mineralized and sound dentin matrices contain inactive preforms of proteolytic enzymes that may be activated during the demineralization cycle. In this study, we tested the hypothesis that protease inhibitors (PI) preserve demineralized collagen fibrils and other constituents of the dentin matrix and thereby affect the potential for remineralization. Artificial carious lesions with lesion depths of 140 μm were created with acetate buffer (pH = 5.0, 66 hours), and remineralized using a polymer-induced-liquid-precursor (PILP) process (pH = 7.4, 14 days) containing poly(aspartic acid) (pAsp) as the process-directing agent. De- and remineralizing procedures were performed in the presence or absence of PI. Ultrastructure and mechanical recovery of demineralized dentin following PILP remineralization were examined and measured in water with atomic force microscopy (AFM) and nanoindentation. Nanomechanical properties of hydrated artificial lesions had a low elastic modulus (E_R_ <0.4 GPa) extending about 100 μm into the lesion, followed by a sloped region of about 140 μm depth where values reached those of normal dentin (18.0–20.0 GPa). Mapping of mineral content by both micro-FTIR and micro x-ray computed tomography correlated well with modulus profiles obtained by nanoindentation. Tissue demineralized in the presence of PI exhibited higher elastic moduli (average 2.8 GPa) across the lesion and comprised a narrow zone in the outer lesion with strongly increased modulus (up to 8 GPa; *p* < 0.05), which might be related to the preservation of non-collagenous proteins that appear to induce calcium phosphate mineral formation even under demineralizing physical-chemical conditions. However, mechanical aspects of remineralization through the elastic modulus change, and the micromorphological aspects with SEM and TEM observation were almost identical with PILP treatments being conducted in the presence or absence of PI. Thus, the application of the protease inhibitors (PI) seemed to be less effective in promoting the remineralization of demineralized dentin.

## Introduction

Extrafibrillar and more importantly intrafibrillar remineralization of collagen type I matrices has been suggested to be critical to restoration of the mechanical properties of dentin [1–3]. Gower and coworkers were the first to achieve intrafibrillar mineralization of a variety of collagen-I substrates using the so-called “polymer-induced liquid-precursor (PILP)” mineralization process [4–7]. Comparable approaches followed, using other polyanionic acids [8, 9]. The PILP process consists of anionic polymer macromolecules poly(aspartic acid), a simple mimic for acidic or phosphorylated non-collagenous proteins, which stabilizes a supersaturated mineralization solution by interacting with calcium and phosphate ions and forming nanodroplets of about 15 to 30 nm in diameter [6, 7]. Recently a similar collagen mineralization process was developed using a cationic polymer that identified maintenance of an equilibrium of osmotic pressure and electroneutrality as the main driving force for ion infiltration into fibrils in the PILP mineralization approach [10].

In our previous study [11], we have applied the PILP process to remineralize 140 μm deep artificial carious lesions. After PILP remineralization, there was complete recovery of mineral content throughout the lesion depth, while nanomechanical testing found the reduced elastic modulus (E_R_) recovered to 50–60% of normal dentin in the severely demineralized outer zone and full recovery of the inner zone. However, if process-directing agent like poly(aspartic acid) are unavailable, recovery of properties occurred only in the sloped inner zone of the lesion, while the outer fully demineralized zone did not show any enhancement at all as using calcium phosphate solutions alone was not able to induce intrafibrillar mineral in collagen [12].

It has been hypothesized that activation of endogenous, bound matrix metalloproteinase (MMP) and cysteine cathepsins during demineralization causes denaturation of collagen fibrils leading to changes in collagen bioactivity and incomplete functional remineralization in the most demineralized regions [11]. Therefore the use of protease inhibitors (PI) was suggested to limit collagen fibril damage and thereby allow improved intrafibrillar mineralization and recovery of both structure and mechanical properties during the PILP re-mineralization process [11]. The hypothesis of this study was that PI preserve demineralized collagen fibrils and other constituents of the dentin matrix and thus improve the recovery of mechanical properties by functional remineralization through the PILP approach. Here we investigated the nanomechanical properties during the demineralization and remineralization process in the absence or presence of PI in relation to structural and compositional changes following PILP treatments.

## Materials and methods

### 2.1. Tooth preparation

The study was approved by Committee on Human Research (CHR), The Human Research Protection Program at UCSF (http://www.research.ucsf.edu/chr). Institutional review board waived the need for written informed consent from the participants as no patient data was collected.

Permanent, fully-formed human third molars were obtained from the UCSF dental hard tissue specimen core according to the approved protocols. Teeth were sterilized by gamma radiation, which did not affect their mechanical properties [13], and stored in deionized water at 4°C until prepared. Dentin blocks measuring 4.5 mm in length and width and 2 mm in thickness were cut from the mid-coronal region of the selected teeth perpendicular to the tubule direction. The sample surfaces were ground with SiC abrasive papers from 320 to 1200 grit, and then polished with aqueous diamond suspensions (Buehler Ltd., Lake Bluff, IL, USA) of 3.0, 1.0, and 0.25 μm particle sizes. Each sample surface was sealed with nail varnish (Revlon Corp., New York, NY, USA) except for a 2.5 × 2.5-mm window.

### 2.2. Lesion formation and remineralization

Each dentin sample was immersed in 40 mL of demineralization solution (2.2 mM CaHPO_4_^.^ 2H_2_O; and 50 mM CH_3_COOH at pH 5.0) for 66 hours to create a 140 μm deep artificial caries lesion [11]. Following lesion development, the samples were rinsed and placed in a 40 mL remineralization solution at 37°C under continuous shaking for 14 days. The remineralization solution contained: 50 mM Tris-buffer with 0.9% NaCl, 0.02% NaN_3_, 4.5 mM CaCl_2_, 2.1 mM K_2_HPO_4_ and 100 μg/mL of 27 kDa poly(aspartic acid) (pAsp) (Alamanda polymers, Huntsville, AL) [11]. The pH of the PILP remineralization system was adjusted to 7.4 by addition of NaOH at room temperature. Both de- and remineralization processes were performed in the absence or presence of proteases inhibitors (PI). The inhibitors were: 2.5 mM benzamidine HCl; 0.5 mM N-ethylmaleimide; 50 mM ɛ-amino-n-caproic acid, and 0.3 mM phenylmethylsufonyl fluoride [14]. According to the incorporation of PI into the de- and remineralization cycles, five experimental groups were formed: (1) demineralization (**DE**); (2) demineralization in the presence of PI (**DEpi**); (3) demineralization followed by remineralization (**DE-REM**); (4) demineralization followed by remineralization in the presence of PI (**DE-REMpi**); (5) demineralization in the presence of PI followed by remineralization in the presence of PI (**DEpi-REMpi**). Next, each sample was embedded with epoxy, ground and polished before analysis by optical microscopy, nanoindentation, micro x-ray computed tomography (MicroXCT^TM^), Fourier transform infrared (FTIR) microspectroscopy, scanning electron microscopy (SEM) and transmission electron microscopy (TEM) observation.

### 2.3. Shrinkage of lesions and nanomechanical recovery evaluation

**Fig 1:**
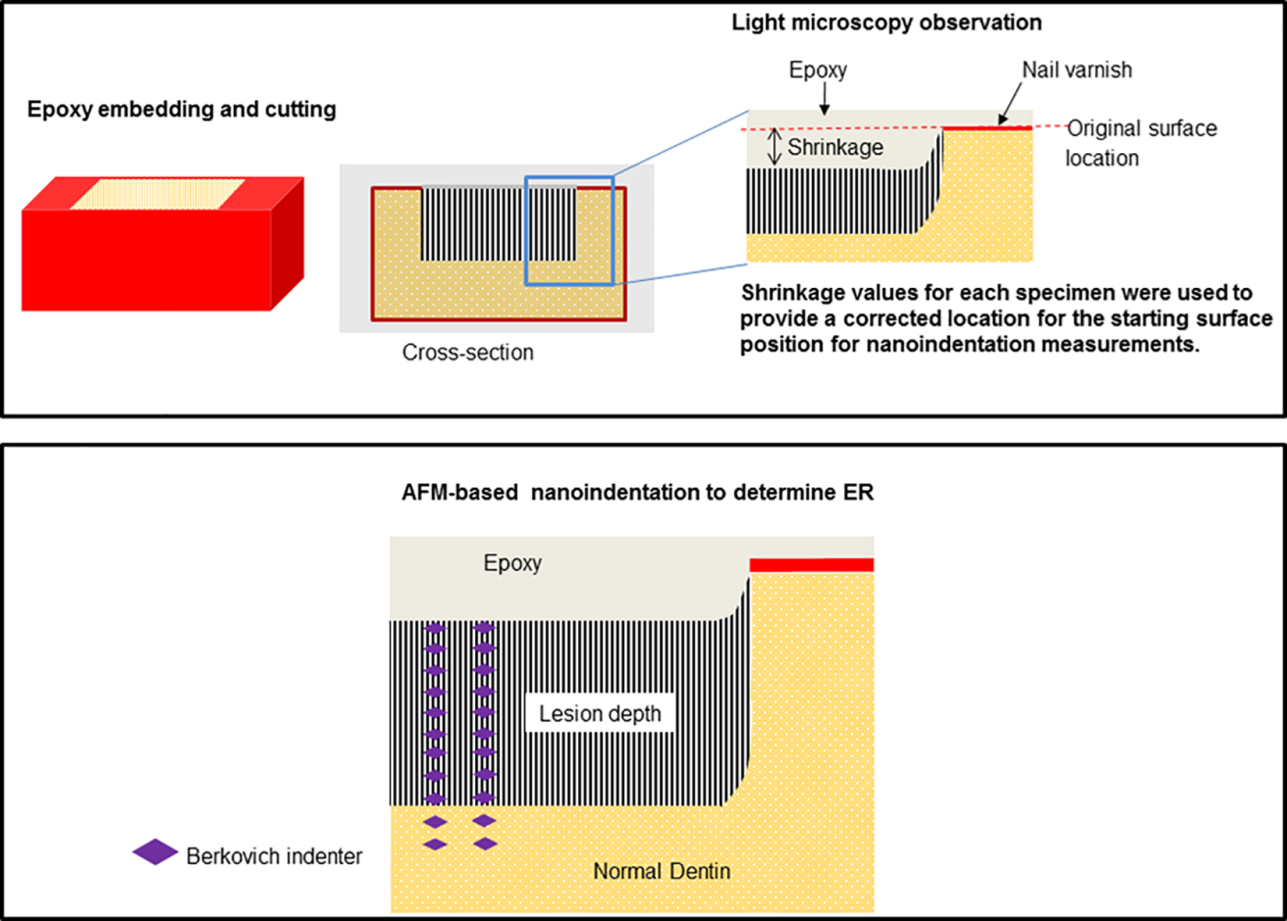
Schematic drawing for the sample preparation and visualization under the light microscopy observation and AFM-based nanoindentation analysis.

The blocks containing the lesions were embedded at room temperature in epoxy (Epoxicure; Buehler, Lake Bluff, IL). After embedding each sample was examined in an optical microscope to determine the amount of shrinkage that occurred when the specimen was dehydrated during embedding and the demineralized/remineralized zone collapsed in areas of insufficient mineralization with reference to the adjacent normal dentin [11]. The individual shrinkage values for each sample were measured in cross sections as a distance from surface of the normal dentin to the surface of the treated zone, e.g. demineralized/remineralized, and used to provide a corrected location for the starting surface position E-modulus plots obtained by nanoindentation (Fig 1).

The blocks were cut perpendicular to the surface to expose the lesion structure from the most demineralized outer portion through the lesion and into sound dentin using a low speed water-cooled diamond saw (Isomet, Buehler). The blocks were cut into two halves, one for nanoindentation and the other for SEM and TEM characterization. The cross sections (minimum thickness of 400 μm) were fixed to metal AFM discs (Ted Pella, Redding, CA) using a cyanoacrylate adhesive (QX-4, MDS Products, Laguna Hills, CA) and prepared for nanoindentation by polishing as described above and hydrated in de-ionized water for one hour before measurements started. “Wet” nanoindentation (n = 5 specimen *per* group) was performed in a liquid cell using an AFM (Nanoscope III Veeco Instruments, Santa Barbara, CA) to which a load-displacement transducer (Triboscope, Hysitron Incorporated, Minneapolis, MN) was attached. A long-shaft diamond Berkovich indenter with a conventional radius of curvature less than 100 nm (Hysitron) was attached to the transducer. Fused silica was used to calibrate the transducer under dry and wet conditions. Site-specific measurements of reduced elastic modulus (E_R_) were made using a controlled force of 200 μN to 500 μN with a 3-second trapezoidal loading profile (load, hold, and unload) [11]. Indentations were made at intervals of 4 μm starting from the most demineralized outer surface of the lesion and proceeding inwards through the depth of the lesion and into sound dentin, covering a total distance of 180 μm. Two similar lines of indents were made in each sample.

To analyze the changes in nanomechanical properties as influenced by each treatment, the reduced E_R_ measurements were plotted against distance from the sample surface, and the area under the curve (AUC) was calculated (GPa-μm). The data was statistically analyzed by one-way ANOVA and Tukey’s multiple comparison tests with statistical significance set at α = 0.05 [15]. The hardness values that were simultaneously obtained for each indentation were highly correlated (data not shown) with values of elastic modulus, and thus were not included for brevity.

### 2.4. MicroXCT^TM^

Following nanomechanical characterization, thin cross sections (~500 μm) from the centers of the dentin samples (n = 1 for each experimental condition) were prepared for analysis using MicroXCT^TM^ (Xradia, Inc., Pleasanton, CA) following a previously described protocol [11, 15] to determine the extent of remineralization. Scanning parameters used were 10X magnification, a peak voltage of 50 kVp, 133 μA, and 2000 image projections, with a pixel size of 1.67 μm. Intensity resulting from x-ray attenuation due to mineral density variation within the experimental region was compared with sound dentin using XMController version 8.2.3724, the data acquisition software for the MicroXCT^TM^, by selecting line profiles starting from the most demineralized outer surface of the lesion and into sound dentin, covering a total distance of 180 μm. Similar lines of regions of interest (ROI) were placed nearby in the dentin samples. Thus each sample contained three lines of intensity profiles, that extended from the outer surface and into sound dentin, that were plotted and compared.

### 2.5. FTIR microspectroscopy

To assess changes in the amount of phosphate present after the de- and remineralization process FTIR microspectroscopy (Nicolet iN10, Thermo Scientific Inc., Waltham, MA, USA) was applied. The dentin samples from nanomechanical characterization (nanoindentation) were used and scanned to obtain spatially resolved FTIR spectra, spanning the region from the top of the demineralized lesion zone (carious dentin) down to the normal zone, at a pixel resolution of 25 μm in the mid-infrared region of 4,000–720 cm^-1^ with 8 scans per pixel. An atmospheric correction was applied to remove the contribution of the atmosphere background. The determination of band area was performed with the OMNIC Picta software (Nicolet) following a two-point baseline correction, band area integration, and deconvolution using the Kramer-Kronig algorithm. The zone between 1160 and 1020 cm^-1^ is associated with two vibrational modes of the phosphate molecules in minerals with peak intensities around 1110 and 1065 cm^-1^. The total area under these peaks was plotted as a function of location on the sample (with approximately 25 μm resolution) and used to produce FTIR maps of mineral content and identification of demineralized and remineralized zones in comparison to the sound dentin surrounding each lesion.

### 2.6. SEM and TEM characterization

Selected samples (n = 2 per group) from each group were evaluated by SEM and TEM to characterize structural variations. For SEM, samples were coated with a 10–20 nm thick Au thin film using a sputter coater (Denton Vacuum Inc., Model # Desk II, Moorestown, NJ) and imaged using a Hitachi S-4300 field emission gun scanning electron microscope (Hitachi High Technologies America, Pleasanton, CA) at an accelerating voltage of 10 kV at working distances of <12 mm. For TEM, samples were further trimmed and embedded after ethyl alcohol and acetone dehydration in Spurr’s resin (Ted Pella, Redding, CA). Unstained, 70- to 80-nm-thick sections were examined by means of a JEM-1400 TEM (JEOL, Tokyo, Japan) at 120 kV following the general procedures described previously [16].

## Results

### 3.1. Optical microscopy and shrinkage analysis

**Fig 2:**
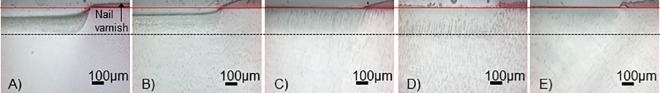
Optical images and shrinkage of dried cross sections exposing the lesion depths. A) demineralized lesion (DE) with nail varnish shown protecting the unexposed surface; B) shrinkage was greatly decreased when demineralized in the present of PI (DEpi) and and was almost undetectable at C) DE-REM; D) DE-REMpi; and E) DEpi-REMpi. Solid red line = original surface location, black dotted line = lesion depth.

Dehydration of dentin leads to a collapse of the collagenous matrix when not supported by apatite mineral or absorbed water. This collapse was visualized on cross sections through the artificial lesions and quantified as the distance from the normal dentin surface to the surface of the collapsed demineralized zone as shown in Fig 2. We have shown previously that after 66 hours of demineralization a total lesion depth of about 140 μm is reached in dentin [17]. This leads to shrinkage of the demineralized zone of 64.8 ± 4.7 μm upon drying (Fig 2A, Table 1). When PI were present during the demineralization process (DEpi), shrinkage was considerably less (Fig 2B) and measured only 28.2 ± 3.2 μm. Remineralization succeeded in reinforcement of the dentin matrix and reduced the shrinkage to 4.2 ± 1.0, 4.1 ± 0.4 and 0.5 ± 0.7 μm for DE-REM, DE-REMpi and DEpi-REMpi, respectively (Fig 2C–2E).

**Table 1:** Average shrinkage measurement.

Treatment Group	Average shrinkage measurement (μm)
A) DE	64.8 ± 4.7
B) DEpi	28.2 ± 3.2
C) DE-REM	4.2 ± 1.0
D) DE-REMpi	4.1 ± 0.4
E) DEpi-REMpi	0.5 ± 0.7

### 3.2. AFM nanoindentation

**Fig 3:**
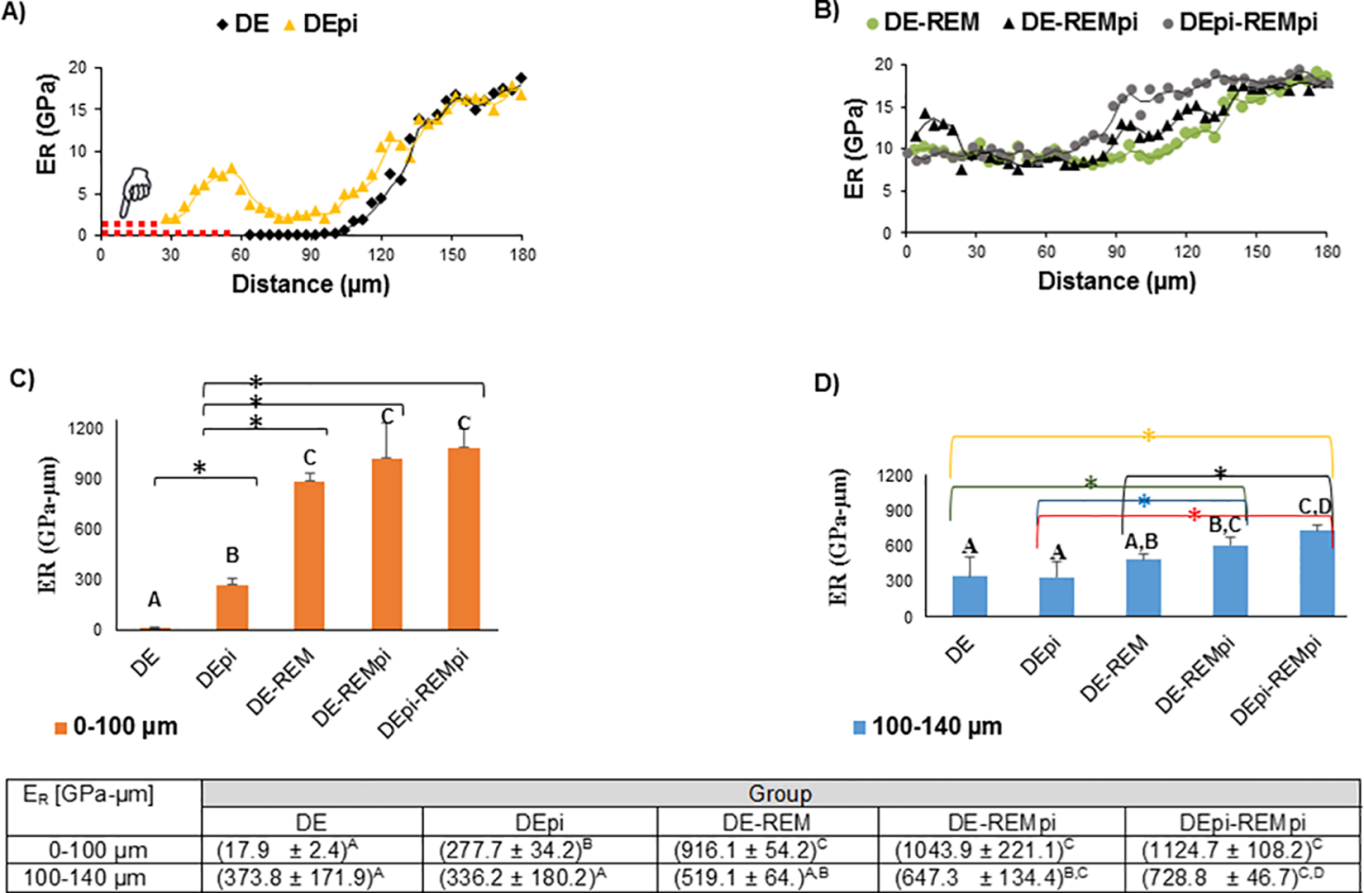
Average elastic modulus (E_R_) values across human dentin from most demineralized outer surface of the lesion and proceeding inwards through the depth of the lesion and into sound dentin before (A) and after PILP treatments (B). Results and statistical comparisons from calculations of the differences in areas under the data curves from 0–100 μm (C) and 100–140 μm (D) depths of each group. Means designated with the different capital letters indicate significant differences between groups, using one-way ANOVA and Tukey's multiple comparison tests (*p < 0.05). Mean ± SD; n = 5; dotted red line = shrinkage (designated by pointer).

Discrete nanoindentations were performed on cross sections along lines across fully hydrated lesions before and after PILP treatments. Fig 3 shows average values of the reduced elastic modulus (E_R_ as a function of distance from the lesion surface). Taking the shrinkage of the lesion zone into account a zone of very low modulus (E_R_ < 0.3 GPa) was observed to a depth of about 100 μm in demineralized lesions followed by a gradual linear increase that reached normal dentin modulus values at a depth of about 140 μm (Fig 3A), in agreement with previous reports [11]. The AUC (GPa-μm; 0–100 μm) and modulus profiles were significantly changed when demineralization was performed in the presence of PI (*p* < 0.05, Fig 3C). Modulus levels did not decrease below 2 GPa across the lesion. In addition a zone of increased stiffness was observed in the outer zone between 40 and 60 μm depth, reaching values of up to 7 GPa (Fig 3A). Even though strict protocol was followed, this increased value was subsequently confirmed upon carefully repeating the evaluation. After remineralization for 14 days in the absence of PI (DE-REM), the characteristic recovery of the outer lesion to values of around 10 GPa was observed (Fig 3B and 3D), as previously described [11]. When PI were added only to the remineralization treatment (DE-REMpi), recovery of modulus was very high but only in the outer 20 μm of the lesion, while the rest of the lesion showed a similar trend of improvement as those results from the other PILP treatments. When the same treatment was performed on lesions that were created in the presence of PI (DEpi-REMpi), the outer lesion recovered to values around 10 GPa as commonly observed with PILP treatments. In addition, normal dentin modulus values were reached at a smaller depth reaching values of about 18 GPa at 80 μm in depth. It is important to note that when comparing the AUC (i.e. 100–140 μm) of the DEpi-REMpi to DE-REM and DE-REMpi, data shows statistically significant differences (p < 0.05, Fig 3D).

### 3.3. MicroXCT^TM^

**Fig 4:**
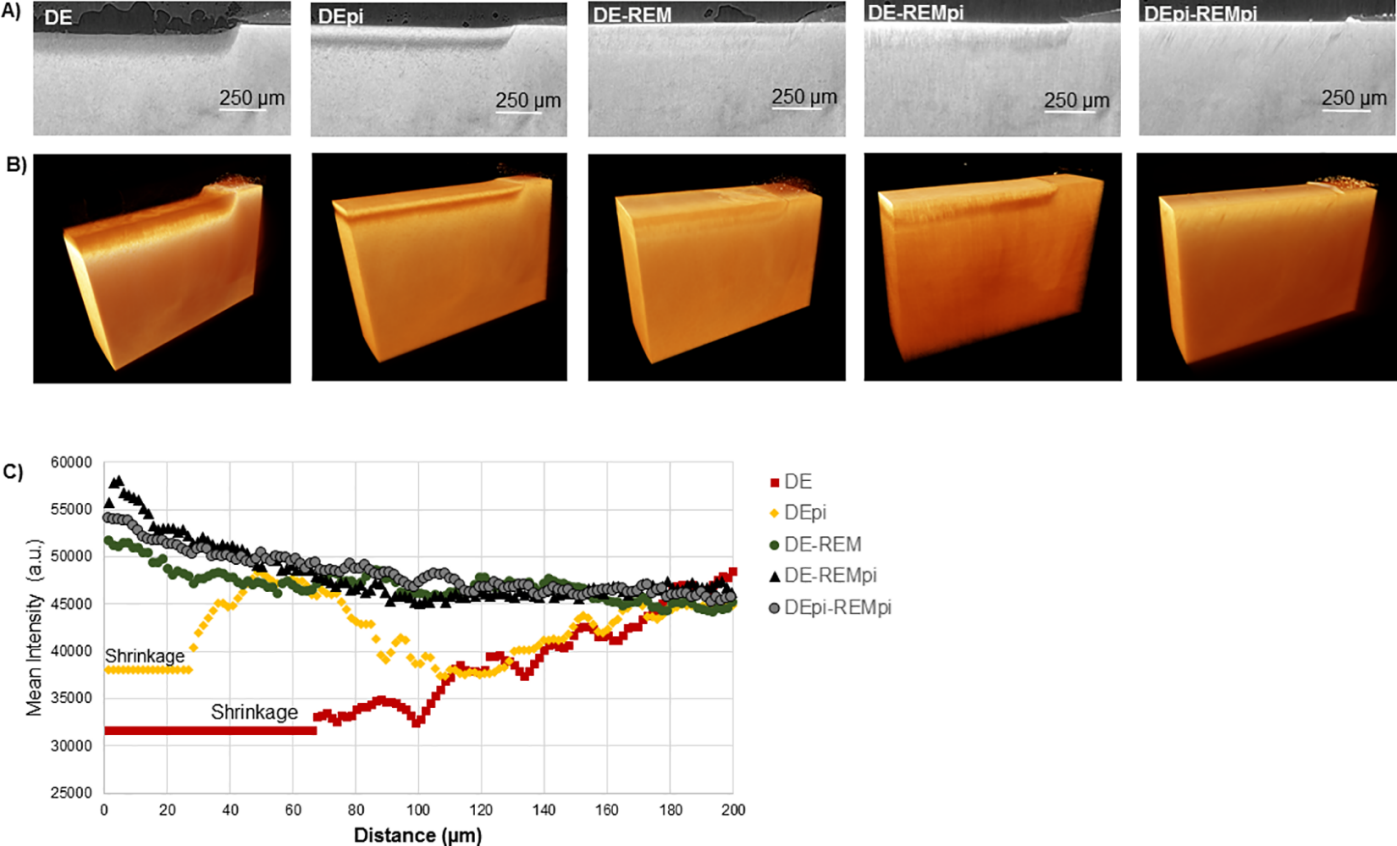
(A) X-ray virtual slices illustrating x-ray attenuation data from MicroXCT tomograms of each sample. **(**B) Three-dimensional plots of attenuation intensities across the samples. (C) Mineral intensity profiles in arbitrary units (a.u.) across human dentin from most demineralized outer surface of the lesion and proceeding inwards through the depth of the lesion and into sound dentin before and after PILP treatments (open circles, squares, triangles, and diamonds).

Tomographic analysis of demineralized dentin slices before and after remineralization were used to establish mineral density profiles based on attenuation changes using arbitrary units (a.u.). Fig 4A shows a cross sectional view through the lesion obtained from MicroXCT^TM^ data. Fig 4B shows three-dimensional plots of attenuation intensities across the samples and Fig 4C is a graph of the attenuation intensity along a line across the lesion for each sample group analyzed. Shrinkage for the demineralized samples without and with PI was 65 and 28 μm, respectively. This is in good agreement with the optical microscopy and nanoindentation data (Figs 2 and 3). In the presence of PI, the total demineralized zone was significantly reduced from the 140 μm of the standard treatment to about 100 μm (DEpi, Fig 4). Dentin was significantly less demineralized when PI were present and showed a substantial amount of mineral, reaching almost normal dentin values in the outer zone of the lesion (Fig 4C). When remineralized by immersion into PILP solutions for 14 days (DE-REM), the mineral level as determined by MicroXCT^TM^ appeared to be fully recovered (Fig 4C). Similarly, when the PILP treatment was performed with additions of PI (De-REMpi) and/or when both the demineralization and the remineralization process were performed in the presence of PI (DEpi-REMpi), the lesion recovered fully to normal intensity values with a slight increase in intensity in the outer zone of the lesion (Fig 4C).

### 3.4. Micro-FTIR analysis

**Fig 5:**
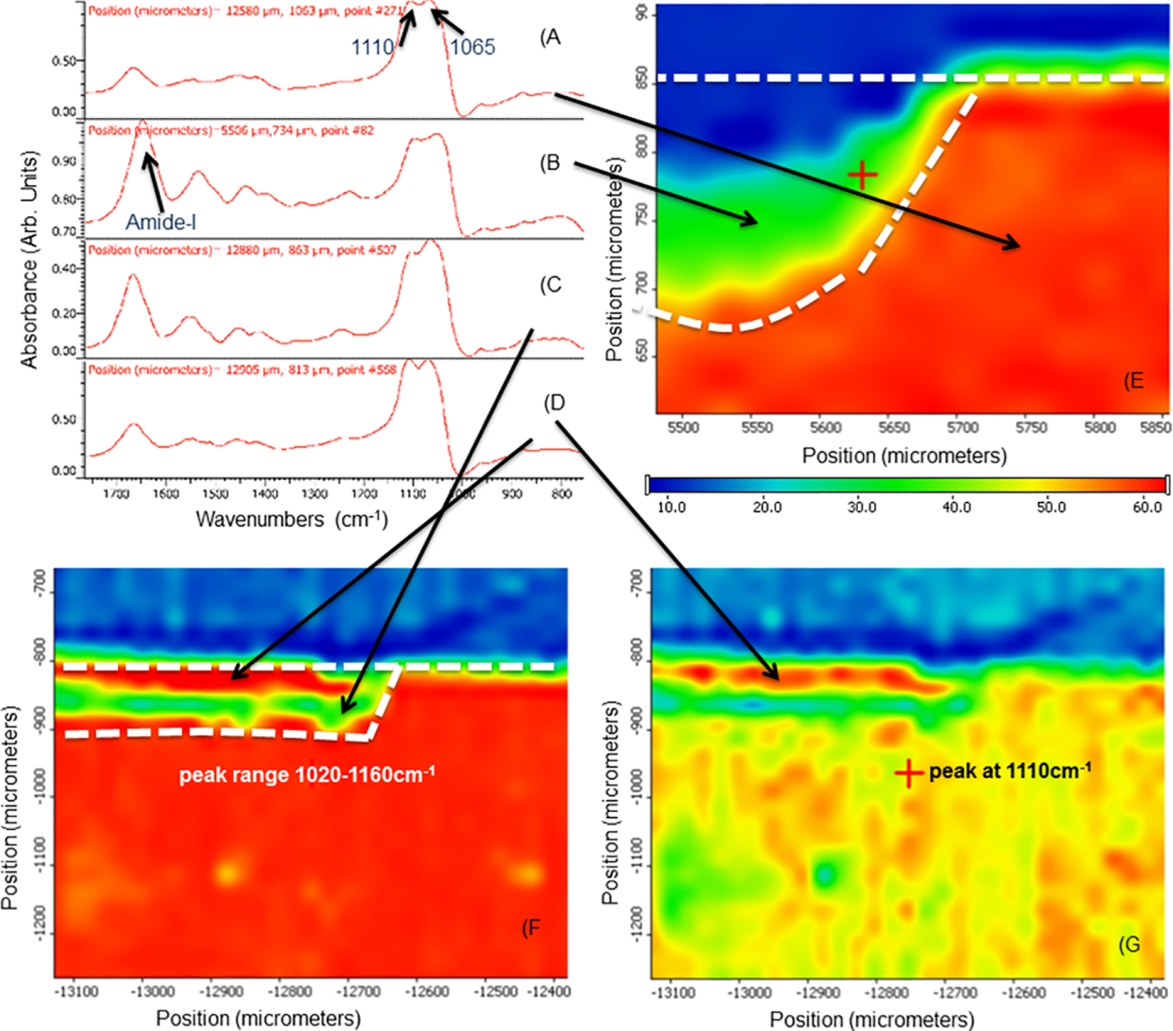
FTIR and micro-FTIR analysis of demineralized and remineralized dentin. A-D) selected individual spectra obtained from area as indicated by arrows, showing peaks associated with phosphate groups at 1020–1160 cm^-1^ and around 1660 cm^-1^ for amide-I; E) micro-FTIR map of cross section through the demineralized dentin lesion (DE), plotted as area of intensity between 1020–1160 cm^-1^; F) micro-FTIR map for range 1020–1160 cm^-1^ of DEpi sample and G) micro-FTIR map of same sample (DEpi) but plotted for intensity of peak at 1110 cm^-1^. Bar shows false color scale to indicate intensity with red being highest and blue lowest.

**Fig 6:**
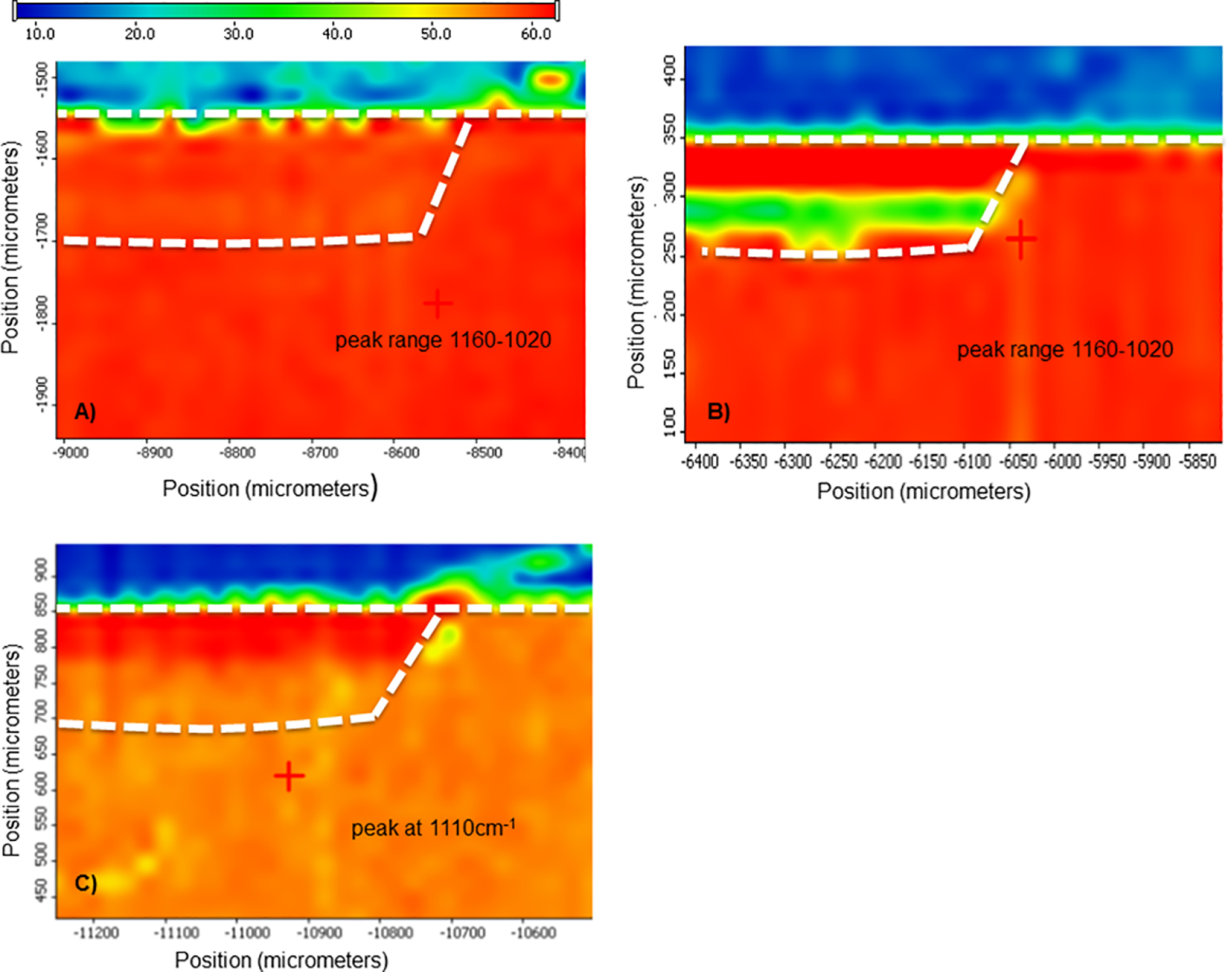
**FTIR maps of remineralized samples of A) DE-REM at 1160–1020 cm^-1^ range; B) DEpi-REMpi at 1160–1020 cm^-1^ range and C) DEpi-REMpi at 1110 cm^-1^, respectively**.

Samples examined by nanoindentation were also used for analysis of mineral concentration by micro-FTIR. Figs 5 and 6 show plots of the area under the phosphate peaks between 1160 and 1020 cm^-1^ (highlighted in Fig 5A) as a function of location on the sample with a spatial resolution of approximately 25 μm. Fig 5A shows a characteristic spectrum between 1800 and 700 cm^-1^ of normal dentin with bands associated with amide I, amide II, C-H and PO_4_ indicated. FTIR maps confirmed that the standard demineralization process produces a zone of about 140 μm depth that is depleted in mineral content. There was a surface collapse of about 60 μm (blue), followed by a zone of low mineral content (green) leading into a transition zone with increasing mineral content (yellow to red) until normal phosphate levels were reached (red) at about 150 μm lesion depth (Fig 5B). FTIR maps of the phosphate region looked quite different when demineralization was performed in the presence of PI (DEpi). The total depth was only approximately 100 μm. A surface region of up to 50 μm depth was comprised of phosphate containing mineral (Fig 5C). The region associated with the phosphate vibration at 1110 cm^-1^ revealed that the surface zone has a larger component of phosphates associated with that vibrational mode indicating that the mineral in this zone is different from the apatite mineral found in sound dentin that surrounds these lesions. FTIR maps of remineralized samples are shown in Fig 6. Fig 6A shows a narrow zone of about 20 μm at the surface of the PILP-treated sample that is somewhat lower in mineral content. Most of the lesion appears to have recovered fully and reached apparently normal mineral content according to the FTIR analysis, similar to the data obtained by MicroXCT^TM^ (Fig 4C). Samples that were remineralized in the presence of PI (DE-REMpi) showed a full recovery of mineral in a superficial zone of about 50 μm width, followed by a zone with lower mineral content of equal width before transitioning quickly into normal dentin (Fig 6B). When both treatments were carried out with PI additives (DEpi-REMpi), FTIR analysis revealed an increased mineral content in the outer zone of the lesion that has increased above the level of normal dentin (Fig 6C); that zone was about 60–70 μm deep. Analysis of this zone revealed that it was primarily comprised of phosphates associated with the 1110 cm^-1^ band versus components associated with the 1065cm^-1^ band.

### 3.5. SEM and TEM characterization

**Fig 7:**
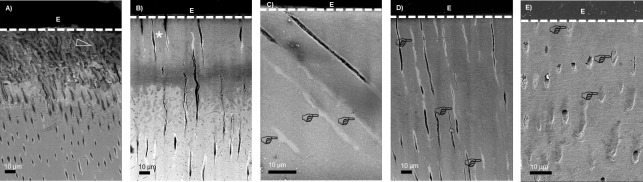
SEM images of the samples after demineralization (A; DE), demineralization in the presence of PI (B; DEpi), demineralization followed by remineralization (C; DE-REM), demineralization followed by remineralization in the presence of PI (D; DE-REMpi), and demineralization in the presence of PI followed by remineralization in the presence of PI (E; DEpi-REMpi). Before PILP-treatment showing the exposed reticular microstructures of the collagen fibrils (open arrowhead). In the DEpi group, the effect of demineralization decreases. Since there was no apparent interfibrillar space, it was difficult to find traces of collagen fibrils in the intertubular dentin (asterisk) area. After PILP-treatment, regardless in the presence or absence of PI, showing electron-dense intrafibrillar and/or extrafibrillar apatite crystals, which were deposited within collagen scaffold. The apparent deposition of the apatite crystals was also found in the intratubular dentin (pointers). E = Epoxy resin.

**Fig 8:**
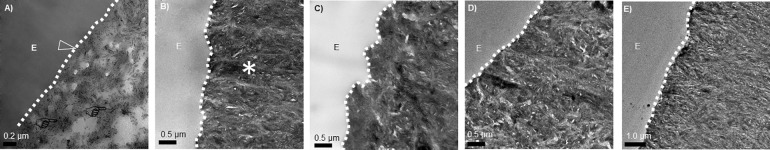
TEM images of the samples after demineralization (A; DE), demineralization in the presence of PI (B; DEpi), demineralization followed by remineralization (C; DE-REM), demineralization followed by remineralization in the presence of PI (D; DE-REMpi), and demineralization in the presence of PI followed by remineralization in the presence of PI (E; DEpi-REMpi). Before PILP-treatment showing the collagenous matrix at the acid-etch outer lesion sites (open arrowhead) was severely demineralized but contained sparsely arranged crystallites (pointers). In the DEpi group, the effect of demineralization decreases and the outer lesion sites had a higher electron density (asterisk) compared with that of the DE group. After PILP-treatment, regardless in the presence or absence of PI, showing electron-dense intrafibrillar and/or extrafibrillar apatite crystals, which were deposited within collagen scaffold. E = Epoxy resin.

Representative SEM images of the initial lesion and remineralization groups are shown in Fig 7, and TEM images are presented in Fig 8. Before PILP-treatment, the DE lesion had a severely demineralized outer region with exposed reticular microstructures of the collagen fibrils created by mineral loss due to the acid challenge (Fig 7A). In the presence of PI (DEpi) group showed a less and mild demineralization. Since there was no apparent interfibrillar space, it was difficult to find a trace of collagen fibrils on the acid-etch lesion sites (Fig 7B). After PILP-treatment, regardless of the presence or absence of PI, specimens had electron-dense intrafibrillar and/or extrafibrillar apatite crystals, which were deposited within collagen scaffold (Figs 7C–7E). The apparent deposition of the apatite crystals was also found in the intratubular dentin.

Under TEM observation, the collagenous matrix of artificial caries lesion sites (DE) was severely demineralized with some residual crystallites remaining (Fig 8A), while DEpi group showed more electron-dense crystals indicating substantial amounts of mineral at the surface of the artificial lesion (Fig 8B). Regardless, in the presence or absence of PI, after 14 days of PILP remineralization, the collagenous matrix showed uniform remineralization characterized by electron-dense intrafibrillar and/or extrafibrillar apatite crystals (Fig 8C–8E).

## Discussion

Biomineralization of collagenous tissues, like bone and dentin, is a complex process involving the secretion, assembly and organization of matrix molecules that predominantly suppress mineral formation. Only after the processing and modification of collagen and non-collagenous proteins (NCPs) will mineral deposition occur and allow for apatite crystals to develop at highly specific nucleation sites with controlled crystal orientation and size [6]. This study sought insights into the role of protease inhibitors (PI) leading to remineralization and mechanical recovery of demineralized dentin using the PILP mineralization system. In this respect, demineralized dentin contains active form of endogenous proteases and collagen with cross-linked bound NCPs which are known to be critical for mineralization [18] and may also be useful for remineralization approaches using chemical treatments like the PILP method.

Initially this study examined the potential for including PI into the PILP mineralization approach as the inhibition of further processing of collagen during the remineralization process was expected to enhance fibril integrity and thus improve recovery of mechanical properties. This hypothesis was not proven correct, as mechanical recovery was almost identical with PILP treatments being conducted in the presence or absence of PI. In addition, the DE-REM (4.2 ± 1.0) and DE-REMpi (4.1 ± 0.4) groups (Table 1) have comparable shrinkage values, which is a reasonable indicator of mineral incorporation within the lesion, suggesting the presence of PI has no significant effect in the remineralization of demineralized dentin. Moreover, no immediate difference between the two treatments was observed by nanoindentation (Fig 3), tomography (Fig 4), spectroscopy (Figs 5–6) or electron microscopy (Figs 7–8) suggesting that, if indeed endogenous proteases cause harm to collagen fibrils, it may already have occurred during the demineralization process and an intervention during the remineralization treatment may be too late. Therefore an additional group of experiments was designed which included PI cocktail during the demineralization process (DEpi-REMpi). This PI cocktail contains phenylmethylsufonyl fluoride to inhibit serine proteinases, N-ethylmaleimide, ɛ-amino-n-caproic acid to inhibit cysteine proteinases and benzamidine HCl which inhibit MMPs and other proteases preventing the processing of collagen fibrils and remnant NCPs [14, 19] that are present in the demineralized dentin matrix [18]. Interestingly, in this group the demineralization profile when evaluated with nanoindentation was quite different. After 66 hours of demineralization the overall lesion depth was reduced by almost 50% when PI were used (Fig 3). In addition, only the inner zone of the lesion below 50 μm depth showed a severe depletion of mineral and significantly reduced properties, which were still higher (E_R_ ~2 GPa) when compared to the outer lesions of the standard demineralized samples (E_R_ ~ 0.2 GPa). All samples examined showed an outer zone of about 50 μm in depth with elevated stiffness and hardness. The presence of mineral in this zone was confirmed by all methods applied, e.g. MicroXCT^TM^ (Fig 4), micro-FTIR (Figs 5–6), SEM (Fig 7), and TEM (Fig 8). In addition, there was a slight peak shift in FTIR spectra from 1065 cm^-1^ to 1110 cm^-1^ when comparing normal dentin to treated dentin. Both absorption bands are associated with phosphate in minerals, and both have been reported as an asymmetric stretching mode in apatite as well as other calcium phosphates like β-tricalcium phosphate (β-TCP) [20]. The change in ratio between the two phosphate bands comparing outer lesion and normal dentin is well illustrated in the FTIR graphs shown in Figs 5 and 6. While it was not possible to clearly identify a different calcium phosphate phase in the outer zone of the lesion, this shift indicates that mineral forming in the outer zone probably formed during the 66 hours of immersion time in the demineralization buffer. The solution prepared for demineralization is slightly undersaturated at pH 5.0. Thermodynamically mineral cannot precipitate under these conditions, however locally one can assume that the continued demineralization process of dentin leads to an increase in calcium and phosphate primarily in the vicinity of the demineralized dentin lesion and within the collagen matrix. Under standard conditions of demineralization without PI we have not observed mineralization to occur. Only in the presence of the inhibitors this mineralized zone developed. At this point we hypothesize that this must be related to activity of the NCPs in the dentin matrix. Due to the presence of PI, NCPs are not further processed and hydrolyzed and can thus continue to act as mineralizing agents. NCPs like dentin matrix protein and dentin phosphoprotein have shown an ability to mineralize collagen [21] and are essential to proper dentin mineralization [15]. The protection from further processing of these molecules and the increase in local saturation in calcium and phosphate may therefore allow for mineral formation in the outer dentin lesion while the demineralization process continued in the deeper zone [11]. Although the exact mechanism of NCPs protection in demineralized dentin is not fully understood. It is generally accepted that the most synthetic protease inhibitors prevent MMPs activity, which has been shown to participate in the degradation of NCPs [22, 23], by chelating or replacing the active-site zinc ion. This metal ion binds to specific sites of the enzyme structure inducing conformational changes activating the enzyme [24]. Remineralization via PILP treatments of dentin that was demineralized in the presence of PI (DEpi-REMpi) had reduced depth of the overall demineralized zone (~100 μm). In contrast to the other samples studied, this zone had increased mineral when remineralized in the presence of PI (Fig 6B and 6C). This increase in mineral did not improve the properties beyond the results obtained with the standard PILP treatments (DE-REM), thus suggesting that most of the additional mineral is not functionally bound to dentin collagen and mainly has developed within the dentin tubules as shown by the SEM analysis (Fig 7).

Conversely, it has been shown that chlorhexidine possesses desirable cysteine proteinase [25] and MMP-inhibitory properties [26, 27]. Many studies have reported that applying a chlorhexidine solution to acid-etched dentin prior to the use of adhesives may promote the stability and remineralization of the exposed collagen fibrils in the hybrid layer [28, 29]. From a clinical perspective, it would be advantageous to be able to preserve demineralized dentin matrix [30, 31], however, the protease inhibitor cocktail used in this study may be toxic and thus not be applicable to routine clinical procedures [14]. The biomimetic remineralization strategy based on the PILP process demonstrates great mechanical recovery of caries-like dentin [11] or faulty hybrid layers [32]. However, adding PI to the remineralization process does not appear to improve the functional recovery of the tissue and may thus not be useful for the process of remineralization of caries lesions.

## Conclusions

This study showed that preservation of matrix proteins during the dentin demineralization process allows for mineralization to occur within the demineralized matrix which may be associated with activity of NCPs that are able to function and induce mineralization due to the presence of PI and locally available calcium and phosphate ions released during the demineralization process. This study showed that supplementation of pAsp comprised PILP mineralization treatments with PI did not improve the overall results on mechanical recovery of the lesion. The outer zones of artificial lesions in dentin recovered to about 60% (E_R_ = 9-11GPa) of the normal dentin modulus values (E_R_ = 18–20 GPa) without and with additions of PI.
